# Anaerobic Ammonium Oxidation: From Laboratory to Full-Scale Application

**DOI:** 10.1155/2013/469360

**Published:** 2013-07-17

**Authors:** Shou-Qing Ni, Jian Zhang

**Affiliations:** School of Environmental Science and Engineering, Shandong University, Jinan 250100, China

## Abstract

From discovery in the early 1990s to completion of full-scale anammox reactor, it took almost two decades to uncover the secret veil of anammox bacteria. There were three milestones during the commercialization of anammox: the development of the first enrichment culture medium, the completion of the first commercial anammox reactor, and the fast start-up of full-scale anammox plant. Till now, the culture of anammox bacteria experienced a big progress through two general strategies: (a) to start up a reactor from scratch and (b) to seed the reactor with enriched anammox sludge. The first full-scale anammox reactor took 3.5 years to realize full operation using the first approach due to several reasons besides the lack of anammox sludge. On the other hand, the first Asian anammox reactor started up in two months, thanks to the availability of anammox seed. Along with the implementation of anammox plants, anammox eventually becomes the priority choice for ammonium wastewater treatment.

## 1. Introduction

Conventional biological nitrogen removal from wastewater usually consists of two steps, nitrification and denitrification. During nitrification process, ammonium is biologically oxidized to nitrate, which is then reduced to nitrogen gas using organic matter as electron donor during denitrification process. When BOD/TKN ratio is low as in many ammonium-rich wastewaters, biodegradable organic matter source must be added to achieve complete denitrification [1, 2]. The operations are rather cost-intensive for both oxygen demand for aerobic nitrification and organic substrates addition for denitrification [2–4]. The surplus sludge generated in conventional biological nitrogen removal process also increases the treatment cost.

Anaerobic Ammonium Oxidation (anammox) is a novel, autotrophic, and cost-effective alternative to the traditional biological nitrogen removal process [5–7]. The existence of the bacteria was first predicted in the 1970s on the basis of thermodynamic calculations. Anammox bacteria oxidize ammonium to nitrogen gas using nitrite as an electron accepter under anoxic conditions, and their growth occurs by carbon dioxide fixation (Table 1) [8].

The discovery of anammox process brought revolutionary changes to conventional biological nitrogen removal from wastewater. Some unique characteristics make anammox process a promising and sustainable technique [9], such as low biomass yield, no need for aeration, and no addition of external carbon sources [10]. While the newly discovered anammox process opens up new possibilities for nitrogen removal from wastewater, the major obstacle for the implementation of anammox is the slow growth rate (*μ*_max⁡_ = 0.065d^−1^, doubling time (*t*_1/2_ = ln⁡2/*μ*_max⁡_) of 11 days) of anammox microorganisms [8, 11], making this process difficult for practical wastewater treatments. Meanwhile, anammox bacteria have been extremely difficult to cultivate in pure culture, even *Candidatus* Brocadia anammoxidans has only been purified to apparent homogeneity by Percoll density centrifugation [12]. In order to fulfill practical application of anammox process, researchers focus on the enrichment of slowly growing anammox bacteria. Many studies were carried out to enrich anammox organisms, either by different methods such as biofilm or granulation, or by all types of reactors. This paper reviews the development of anammox process and relative studies in the laboratory, especially the discovery and biochemistry of the bacteria responsible for anaerobic ammonium oxidation. Special attention was paid on the commercialization and full-scale application of anammox technique.

## 2. Discovery and Phylogeny of Anammox

**Figure 1:**
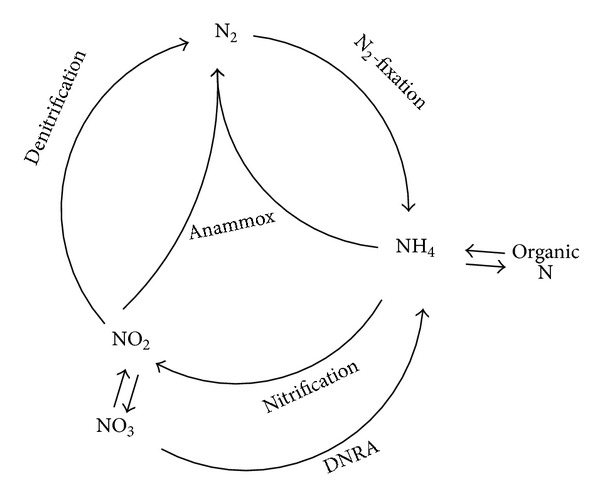
The biological N cycle (based in part on Arrigo [13]). DNRA, dissimilatory nitrate reduction to ammonium.

Already in 1932, it was reported that dinitrogen gas was generated via an unknown mechanism during fermentation in the sediments of Lake Mendota, Wisconsin, USA [14]. More than 40 years ago, Richards [15] noticed that most of the ammonium that should be produced during the anaerobic remineralization of organic matter was unaccounted for. As there was no known biological pathway for this transformation, biological anaerobic oxidation of ammonium received little further attention [13]. Three decades ago, the existence of two chemolithoautotrophic microorganisms capable of oxidizing ammonium to dinitrogen gas was predicted on the basis of thermodynamic calculations [7]. It was thought that anaerobic oxidation of ammonium would not be feasible, assuming that the predecessors had tried and failed to establish a biological basis for those reactions [16]. By 1990s, Arnold Mulder's fantastic observations were just consistent with Richards' suggestion [16]. In their anoxic denitrifying pilot reactor, ammonium disappeared at the expense of nitrite with a clear nitrogen production. The reactor used the effluent from a methanogenic pilot reactor, which contained ammonium, sulphide and other compounds, and nitrate from a nitrifying plant as the influent. This process was named “anammox,” and people realized that it had great significance in the removal of unwanted ammonium. Even without full understanding of anammox reaction, Arnold Mulder patented the process immediately [17, 18]. The discovery of anammox process was first publicly presented at the 5th European congress on biotechnology [19]. By the mid-1990s, the discovery of anammox in the fluidized bed reactor was published [20]. A maximum ammonium removal rate of 0.4 kg N/m^3^/d was achieved. It was shown that for every mole of ammonium consumed, 0.6 mol of nitrate was required, resulting in the formation of 0.8 mol of N_2_ gas ((1) in Table 1). In the same year, the biological nature of anammox was identified [21]. Labeling experiments with ^15^NH_4_^+^ in combination with ^14^NO_3_^−^ showed that ^14-15^N_2_ was the dominant product, making up 98.2% of the total labeled N_2_. These findings conflicted with reaction 1 in which the percentage of ^14-15^N_2_ and ^15-15^N_2_ in the formed dinitrogen gas would be 75% and 25%, respectively. It was realized that, instead of nitrate, nitrite was assumed as the oxidizing agent of ammonium in anammox reaction ((2) in Table 1) [21]. Based on a previous study, Strous et al. [8] calculated the stoichiometry of anammox process by mass balancing ((3) in Table 1), which is widely accepted by other groups. Later, anammox bacteria were identified as planctomycetes [12], and the first identified anammox organism was named *Candidatus* “Brocadia Anammoxidans” [22]. Before 2002, anammox was assumed to be a minor player in the N cycle within natural ecosystems [23]. In 2002, anammox was found to play an important part in the biological nitrogen cycle, accounting for 24–67% of the total N_2_ production in the continental shelf sediments that were studied [24]. Globally, anammox may be responsible for 30–50% of N_2_ production in the ocean [25]. The discovery of anammox process modified the concept of biological nitrogen cycle as depicted in Figure 1.

**Figure 2:**
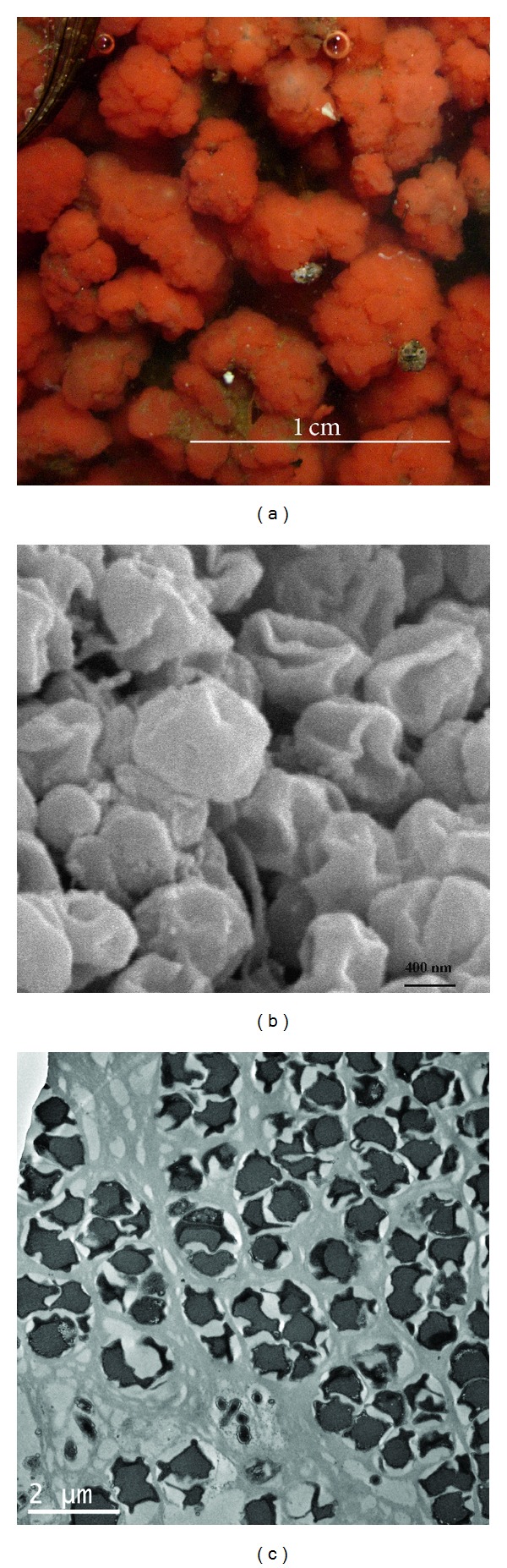
The specific red color of anammox bacteria (a), the typical irregular shapes of anammox bacteria displayed by scanning electron microscopy (b), and transmission electron microscopy images (c).

The specific red color of anammox bacteria (Figure 2(a)) is due to the heme c group of the protein cytochrome c that plays an important role in anammox metabolism [27]. The irregular shapes of anammox bacteria were displayed by both transmission electron microscopy and scanning electron microscopy images (Figures 2(b) and 2(c)). The anammox species have a single membrane bound anammoxosome and riboplasm with ribosome-like particles separated from paryphoplasm by an intracytoplasmic membrane. The cells contain three distinct membrane bound compartments: the paryphoplasm, cytoplasm, and anammoxosome. 

Till now, five anammox genera have been discovered, with 16S rRNA gene sequence identities of the species ranging from 87 to 99% [27]. It is well known that all anammox bacteria belong to the same monophyletic order named the Brocadiales and are related to the Planctomycetales. Among them, four “*Candidatus” *anammox genera have been enriched from activated sludge: “Kuenenia” [28, 29], “Brocadia” [12, 22, 30], “Anammoxoglobus” [31], and “Jettenia” [32]. The fifth anammox genus, “*Candidatus *Scalindua” [33–35], has often been detected in natural habitats, especially in marine sediments and oxygen minimum zones [36–39].

## 3. Possible Reaction Mechanisms for Anammox

**Figure 3:**
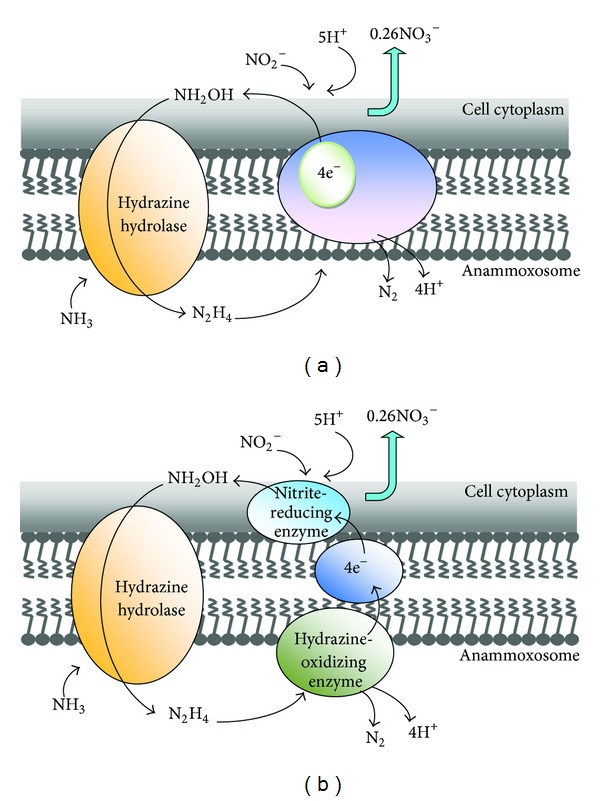
Possible biochemical pathway and cellular localization of the enzyme systems involved in anammox reaction. Figure modified, with permission, from FEMS Microbiology Reviews [26] and Process Biochemistry [2].

To understand the possible metabolic pathway for anammox, ^15^N labeling experiments were first carried out in 1997 [42]. These experiments showed that ammonium was biologically oxidized with hydroxylamine, most likely derived from nitrite, as the probable electron acceptor. The conversion of hydrazine to dinitrogen gas is postulated as the reaction generating the electron equivalents for the reduction of nitrite to hydroxylamine. Generally, two possible reaction mechanisms were addressed [26]. A membrane-bound enzyme complex converts ammonium and hydroxylamine to hydrazine first, followed by the oxidation of hydrazine to dinitrogen gas in the periplasm. At the same time, nitrite is reduced to hydroxylamine at the cytoplasmic site of the same enzyme complex responsible for hydrazine oxidation with an internal electron transport (Figure 3(a)). Another possible mechanism for anammox process is concluded as follows: ammonium and hydroxylamine are converted to hydrazine by a membrane-bound enzyme complex, hydrazine is oxidized in the periplasm to dinitrogen gas, and the generated electrons are transferred via an electron transport chain to nitrite reducing enzyme in the cytoplasm where nitrite is reduced to NH_2_OH (Figure 3(b)). Whether the reduction of nitrite and the oxidation of hydrazine occur at different sites of the same enzyme (Figure 3(a)) or the reactions are catalyzed by different enzyme systems connected via an electron transport chain (Figure 3(b)) remains to be investigated. The occurrence of hydrazine as an intermediate in microbial nitrogen metabolism is rare [43]. Hydrazine has been proposed as an enzyme-bound intermediate in the nitrogenase reaction [44].

**Figure 4:**
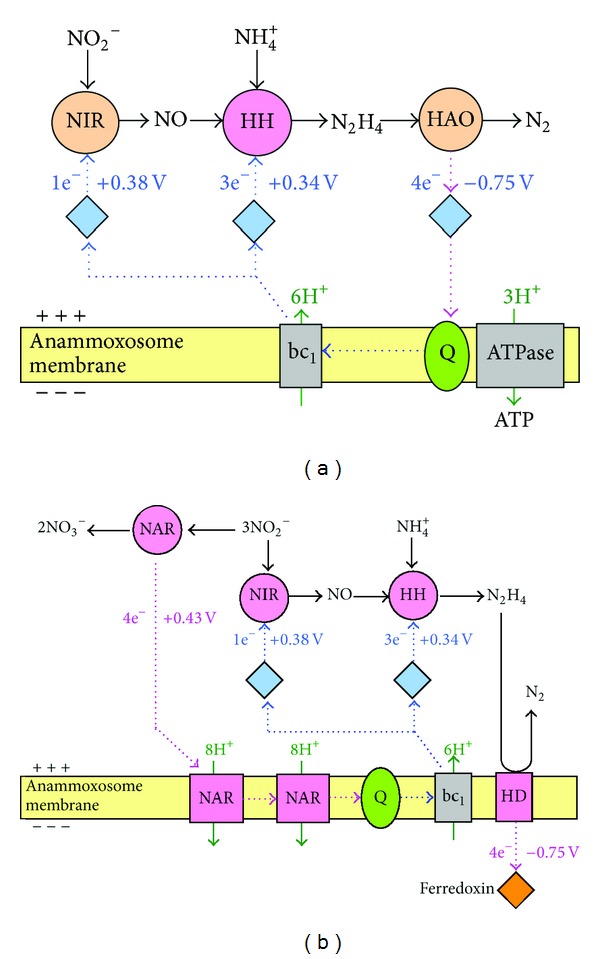
Hypothetical metabolic pathways and reversed electron transport in the anammoxosome. (a) Anammox catabolism that uses nitrite as the electron acceptor for the creation of a proton motive force over the anammoxosomal membrane. (b) Proton motive force-driven reversed electron transport combines central catabolism with nitrate reductase (NAR) to generate ferredoxin for carbon dioxide reduction in the acetyl-CoA pathway. HAO, hydrazine oxidoreductase; HD, hydrazine dehydrogenase; HH, hydrazine hydrolase; NIR, nitrite oxidoreductase; Q, quinine. Light blue diamonds, cytochromes; blue arrows, reductions; pink arrows, oxidations. Figure modified, with permission, from *Nature* [29].

A possible role of NO or HNO in anammox was proposed by Hooper et al. [45] by way of condensation of NO or HNO and ammonium on an enzyme related to the ammonium monooxygenase family. The formed hydrazine or imine could thereafter be converted by the enzyme hydroxylamine oxidoreductase to dinitrogen gas, and the reducing equivalents produced in the reaction are required to combine NO or HNO and ammonium or to reduce nitrite to NO. Environmental genomics analysis of the species *Candidatus* Kuenenia stuttgartiensis, through a slightly different and complementary metabolism mechanism, postulated NO to be the intermediate instead of hydroxylamine (Figure 4) [29]. But this hypothesis also agreed that hydrazine was an important intermediate in the process. In this pathway (Figure 4), there are two enzymes unique to anammox bacteria: hydrazine hydrolase (hh) and hydrazine dehydrogenase (hd). The hh produces hydrazine from nitric oxide and ammonium, and hd transfers the electrons from hydrazine to ferredoxin. Few new genes, such as some known fatty acid biosynthesis and S-adenosylmethionine radical enzyme genes [29], containing domains involved in electron transfer and catalysis were detected.

## 4. Basal and Designated Medium Development

Once nitrite was realized to be the electron acceptor with ammonium as electron donor, a basal medium containing ammonium, nitrite, bicarbonate, minerals, and trace elements was developed for the enrichment of anammox microorganisms [46]. The medium contained ammonium (5–30 mM) and nitrite (5–35 mM), as the only electron donor and electron acceptor, respectively, with bicarbonate (10 mM) as the only carbon source. Minerals and trace elements were also provided. Phosphate concentration of the medium was kept below 0.5 mM, in order to avoid its possible inhibitory effect on the process, and medium was flushed with argon gas to achieve anaerobic conditions. Experiments which were carried out in a fluidized bed reactor with basal enrichment medium showed that the anaerobic ammonium removal rate increased from original 0.4 kg N/m^3^/day to 2.4 kg N/m^3^/day [20]. The maximum specific activity of the biomass in the fluidized bed reactor was 25 nmol NH_4_^+^/mg VS/min. For every mol of ammonium oxidized, 0.041 mol of CO_2_ was incorporated into biomass. The estimated growth rate in the fluidized bed systems was 0.001/h, equivalent to a doubling time of about 29 days. The basal medium enhanced the activities of anammox bacteria.

The development of the basal medium, the milestone of anammox enrichment, turned on the fervent zeal for this infant investigation. Since then, vast number of researchers flooded in this specific topic. As medium shows positive effects on anammox process, many studies focused their attention on this area. Unfortunately, there is no systemic medium development study like those for other bacteria [48, 49].

In our lab, a study was conducted towards designing an appropriate medium by investigating growth requirement of anammox bacteria with respect to amino acids. Twenty L-amino acids were added to basal medium (Table 2). After experiment set I, set II was carried out to further evaluate the enhanced effects of the selective amino acids on microorganisms growth. To quantify the growth of anammox bacteria, quantitative molecular techniques were employed. Preliminary experiments indicated that glycine, methionine, threonine, tryptophan, and tyrosine enhanced the growth of anammox bacteria. On the other hand, asparagine, aspartic acid, and histidine slightly decreased bacterial activities. While 12 of 20 L-amino acids (alanine, arginine, cysteine, glutamic acid, glutamine, isoleucine, leucine, lysine, phenylalanine, proline, serine, and valine) totally inhibited the growth of anammox bacteria, resulting in the sludge turning from reddish to blackish. Another 3 amino acid (asparagine, aspartic acid, and histidine) slowed down the growth of anammox bacteria. This unpublished study would benefit anammox study and their application. 

## 5. Anammox Culture in the Laboratory

Anammox process has been recognized as being difficult to apply for practical applications. Anammox bacteria grow in a mixture of bacterial populations, and they have not been isolated in a pure culture [50]. Anammox bacteria, being strictly anaerobic and autotrophic, are difficult to enrich making application of this process limited due to unavailability of sufficient biomass required for the process. Different methods have been employed to culture and enrich anammox biomass from different types of seed sludge [51, 52]. A relative population of 88% anammox bacteria was achieved in a batch study inoculated from a rotating biological contactor (RBC) treating a landfill leachate [53]. Enrichment culture of anammox bacteria was also developed in lab-scale reactors inoculated with marine sediments [35] and paddy field soil samples and activated sludge from wastewater treatment plants [54]. 

The slow growth rate of anammox bacteria with the approximate doubling time of 11 days is the major obstacle for implementation of anammox process [8]. A long start-up period is thus expected in anammox process. Shortening anammox process start-up period by reducing wash-out potential of anammox biomass becomes an important strategy for full-scale application. Different types of reactor design have been used to minimize the washout of anammox biomass including continuous stirred-tank reactor, anaerobic biological filtrated reactor, sequencing batch reactor (SBR), upflow reactor, and biofilm reactor [8, 55–57]. Faster growth of anammox bacteria was achieved in a membrane bioreactor (MBR) (the doubling time was less than 10 days), resulting in an unprecedented purity of the enrichment of 97.6% [58]. The formation of compact aggregates was reported to maintain a large amount of active anammox biomass in a reactor [55]. Therefore, granulation is also an alternative approach for anammox enrichment. 

**Figure 5:**
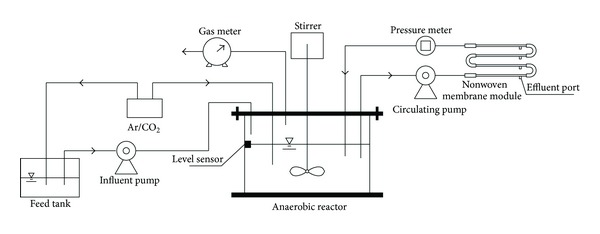
Schematic diagram of the anammox nonwoven membrane reactor (ANMR) [40, 41].

**Figure 6:**
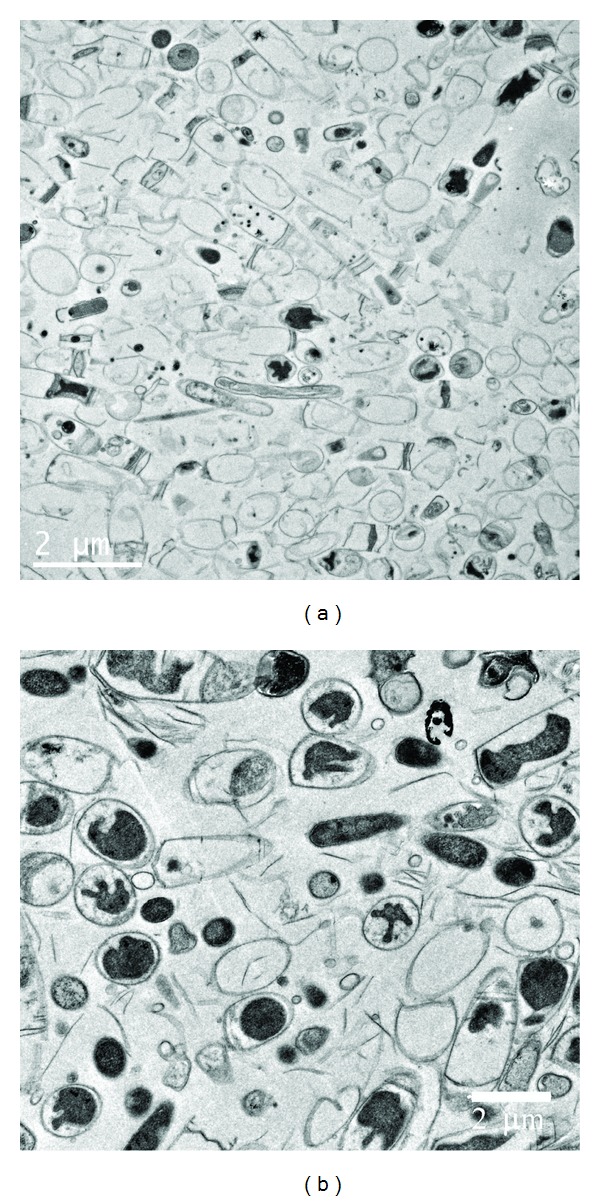
(a) Transmission electron micrograph showing dormant cells in the seed granule (bar = 2 *μ*m). (b) Transmission electron micrograph showing the anammox bacteria in the interior of granules (bar = 2 *μ*m).

In summary, there are two main approaches (strategies) to start up an anammox reactor: (a) to start a reactor from scratch and (b) to inoculate it with highly enriched anammox sludge. For the first strategy, the reactor configuration is very important. The SBR technique ensured over one year reliable operation under stable conditions with efficient biomass retention (more than 90% of the biomass was maintained in the reactor) and homogeneous distribution of substrates, products, and biomass aggregates [8]. The MBR was also applied successfully for cultivation of anammox bacteria with fast growth rate (the minimum doubling time for anammox bacteria was estimated to be 5.5–7.5 days) [58]. Among different reactors, the anammox nonwoven membrane reactor (ANMR) is a novel reactor configuration to enrich anammox biomass (Figure 5) [40, 41]. The reactor was developed by connecting a set of nonwoven membrane module, which also served as an effluent port, with an anaerobic reactor. The membrane module was installed outside the reactor, which is different from the immerged membrane reactors. Unlike conventional MBR, wastewater circulated in the membrane module, and the biofilms grew on the membrane interior surface. A large amount of the suspended biomass could remain in the reactor by filtration through the nonwoven membrane and biofilms, resulting in improvement of the effluent quality and enhancement of the solid retention in the reactor. After over eight months of operation, the purity (percentage of anammox cells in the community) of anammox bacteria in the reactor was quantified to be 97.7% [40]. The cost-effective ANMR was shown to be suitable for the slowly growing anammox bacteria having the following advantages: (1) a large amount of the biomass could remain in the reactor by filtration through the nonwoven membrane and the formation of biofilm, (2) the formation of aggregates and biofilm enhanced the solid retention in the reactor, (3) the nonwoven membrane was cost efficient, and (4) the design of the anaerobic reactor could dilute the influent medium and avoid inhibition from high nitrite concentrations, leading to high tolerance ability of substrates. Recently, the upflow anaerobic sludge blanket (UASB) reactor was highly recommended for the culture of slowly growing bacteria [59–62]. This is because of not only the improvement of physiological conditions, making them favorable for bacteria and their interactions, especially syntrophisms in the anaerobic system, but also the formation of granular sludge, being the major reason of the successful introduction of the UASB reactor [63]. Hence, granulation also improves anammox application. Surprisingly, Ni and his colleagues used inactive methanogenic granules as inocula to realize fast granulation successfully [64]. The start-up nitrite concentration was significantly higher than the published toxic level for anammox bacteria and other lab-scale studies. The accommodations and proliferations of anammox bacteria in the inactive methanogenic granules might be the main reason for the high anammox purity in a short period. Anammox cells could use the skeleton of inactive methanogenic granules and proliferate from the interior as observed in TEM (Figure 6). The second approach mentioned previously significantly shortens the required time for anammox start-up under the premise of large quantity of anammox sludge but is usually limited by the lack of anammox sludge. The gradual construction of full-scale anammox plants increases the availability of anammox sludge. The introduction of the exotic anammox sludge to seed a granular reactor is a good choice [59]. The reactor was started successfully in two weeks; in addition, high nitrogen removal was achieved for a long period, showing that the inoculation of mature anammox granules was ideal to start up a new reactor.

## 6. Commercial Application of Anammox Process

The lack of pure cultures of anammox bacteria makes a genomic approach less straightforward. Combined with the low maximum specific growth rate of anammox bacteria and stringent operational conditions, the practical application of anammox fell far behind the research progress.

Many efforts have been made on the development of a marketable product. Here, we would like to mention the Paques BV (Balk, The Netherlands) for its unremitting efforts on the practical application of anammox process. Early in 2001, Van Dongen et al. [57] scaled up lab-scale SHARON (single reactor system for high rate ammonium removal over nitrite) reactor [3] in collaboration with the Paques BV. The effluent of the SHARON process was ideally suited as influent for anammox process, for the ammonium was oxidized by 53% to nitrite, rather than nitrate in SHARON process at 1.2 kg N load per m^3^ per day without pH control [57]. The combined SHARON-anammox system could work stably over long periods, and the authors predicted that the combination process was ready for full-scale implementation. 

Based on constant and successful study, in 2007, the first full-scale granular anammox reactor was accomplished at the wastewater treatment plant of Waterboard Hollandse Delta in Rotterdam, The Netherland [9, 65]. This stands for the start of the commercial application of anammox process, exhibiting to be another milestone. The first full-scale 70 m^3^ reactor was directly scaled up 7000-fold from 10 l lab-scale experiment. The reactor was initially inoculated with nitrifying sludge and a total amount of 9.6 m^3^; settled biomass from an anammox enrichment reactor was added from day 622 to 1033 [65]. Even with the addition of anammox sludge, the start-up took 3.5 years, 1.5 years longer than designed. Several reasons caused the long start-up time, besides the low growth rates of anammox microorganisms. Most important is that there was no anammox seed sludge available to inoculate the first full-scale reactor, and delay was caused by technical issues such as operational and temperature problems [9], as the first full-scale reactor was directly scaled up from lab scale, skipping the pilot phase. This first full-scale reactor on the other hand had a pilot plant character. In September 2006, the reactor was in full operation and the loading rate could be reached to a level of 750 kg/d, 50% higher than the design load. 

Another four anammox plants were built before 2008, three in Europe and one in Asia (Table 3). The third reactor, part of a plant for the treatment of the effluent of a potato factory, exhibited a largest ammonium load rate. The capacity of the reactor is 1200 kg N/d, while only about 700 kg N/d is converted as no more nitrogen available in the wastewater. Japan built the first full-scale Asian anammox reactor at a semiconductor plant. In 2009, Paques Environmental Technology (Shanghai) released the news that an agreement had been reached to build world's largest anammox based wastewater treatment plant in China. Anammox process was designed to have a capacity for conversion of 11 tons of nitrogen per day, almost ten times larger than the largest plant built before 2008. The two-step combination of anammox and internal circulation (IC) reactors will be the sixth full-scale application of anammox. Since 2009, anammox experienced huge development. Another 11 anammox plants were implemented by Paques, seven of which are located in China. As the world's biggest developing market, China contributes significantly towards commercialization of anammox process.

Thanks to the experience from the established anammox plants, the start-up time of the marketable plant became shorter and shorter. This could be another milestone. The second reactor started up in 1 year and it took 2 months for the start-up of the first Asian plant. Till now, more than 30 full-scale variant plants are in operation around the world, mostly in the Austria, China, Japan, The Netherlands, and USA. All these emphasize on anammox process becoming a commercial technique. 

## 7. Conclusion

 The discovery of the green process, anammox, brings revolutionary changes to conventional biological nitrogen removal. Playing an important part in the biological nitrogen cycle, this unique process makes great contribution to our environment and economy. Anammox development experienced several important points: laboratory culture based on basal medium, full-scale reactor system implementation, and extensive engineering applications. Although starting up the reactor from scratch is universal, inoculation with highly enriched anammox sludge is more feasible. Currently, at least 30 full-scale anammox systems are operational. Thus, application of anammox process offers an attractive alternative to current wastewater treatment systems for ammonia-nitrogen removal.

## Figures and Tables

**Table 1:** Reactions involved in the realization of anammox process.

Reaction no.	Reaction	ΔG°′ (kJ/mol NH_4_^+^)	N_2_ composition (%)	
			^ 14-15^N_2_	^ 15-15^N_2_
1^a^	5NH_4_^+^+ 3NO_3_^−^ → 4N_2_ + 9H_2_O + 2H^+^	−297	75	25
2^a^	NH_4_^+^ + NO_2_^−^ → N_2_ + 2H_2_O	−358	100	0
3^b^	NH_4_^+^ + 1.32NO_2_^−^ + 0.066HCO_3_^−^ + 0.13H^+^ → 1.02N_2_ + 0.26NO_3_^−^ + 0.066CH_2_O_0.5_N_0.15_ + 2.03H_2_O	−358	100	0

^a^Van de Graaf et al. [21].

^b^Strous et al. [8].

**Table 2:** Growth of anammox bacteria using basal medium with L-amino acids.

Amino acid	Plate concentration (mmol/L)	OD_600_*
Alanine	0.5	n.d.
Arginine	0.6	n.d.
Asparagine	0.3	−
Aspartic acid	0.3	−
Cysteine	0.3	n.d.
Glutamic acid	5.0	n.d.
Glutamine	5.0	n.d.
Glycine	0.1	+
Histidine	0.1	−
Isoleucine	0.3	n.d.
Leucine	0.3	n.d.
Lysine	0.3	n.d.
Methionine	0.3	+
Phenylalanine	0.3	n.d.
Proline	2.0	n.d.
Serine	4.0	n.d.
Threonine	0.3	+
Tryptophan	0.1	+
Tyrosine	0.1	+
Valine	0.3	n.d.

*Optical density (600 nm) after 7 days of incubation at 35°C, + means increase, − means decrease, and n.d. means not detected because of the color change.

**Table 3:** The brief description of worldwide full-scale anammox plants implemented by Paques^a^.

Process	Place	Influent	Reactor volume (m^3^)	Designed load (kgN/d)	Year
SHARON-anammox	Rotterdam, NL	Reject water	72	490 (750)^b^	2002
Nitrification-anammox	Lichtenvoorde, NL	Tannery	100	325 (150)^c^	2004
Anammox	Olburgen, NL	Potato processing	600	1200 (700)^c^	2006
Nitrification-anammox	Mie prefecture, JP	Semiconductor	50	220 (220)^b^	2006
Anammox	Niederglatt, Switzerland	Reject water	180	60 (60)^b^	2008
Anammox	Tongliao, China	Monosodium glutamate (MSG)	6600	11000	2009
Anammox	Yichang, China	Yeast production	500	1000	2009
Anammox	Tongliao, China	MSG	4100	9000	2010
Anammox	The Netherlands	Reject water	425	600	2010
Anammox	Tai'an, China	Corn starch and MSG	4300	6090	2011
Anammox	Poland	Distillery	900	1460	2011
Anammox	Wuxi, China	Sweetener	1600	2180	2011
Anammox	Wujiaqu, China	MSG	5400	10710	2011
Anammox	Coventry, UK	Reject water	1760	4000	2011
Anammox	Shaoxing, China	Distillery	560	900	2011

^a^Abma et al. [47] and communication with Paques BV.

^b^Values in parentheses mean achieved loads (kg N/d).

^c^No more nitrogen available.
